# The Added Value of Digital Body Chart Pain Surface Assessment as an Objective Biomarker: Multicohort Study

**DOI:** 10.2196/62786

**Published:** 2025-04-16

**Authors:** Maxime Billot, Amine Ounajim, Maarten Moens, Lisa Goudman, Jean-Philippe Deneuville, Manuel Roulaud, Kévin Nivole, Mathilde Many, Sandrine Baron, Bertille Lorgeoux, Bénédicte Bouche, Lucie Lampert, Romain David, Philippe Rigoard

**Affiliations:** 1 PRISMATICS Lab CHU de Poitiers Poitiers France; 2 Centre de Recherche sur la Cognition et l’Apprentissage Université de Poitiers; Université François Rabelais de Tours; CNRS Poitiers France; 3 Department of Neurosurgery Universitair Ziekenhuis Brussel Brussels Belgium; 4 STIMULUS Consortium Vrije Universiteit Brussel Brussels Belgium; 5 Department of Radiology Universitair Ziekenhuis Brussel Brussels Belgium; 6 Foundation Flanders - FWO Brussels Belgium; 7 Service de Neurochirurgie du Rachis, Chirurgie de la Douleur et du Handicap CHU de Poitiers Poitiers France; 8 Service de Médecine Physique et Réadaptation CHU de Poitiers Poitiers France; 9 Pprime Institute Université de Poitiers CNRS, ISAE-ENSMA Poitiers France

**Keywords:** chronic pain, neuropathic pain, mechanical pain, assessment tool, digital body chart, pain assessment, pain treatment, digital tool, quality of life, financial burdens, machine learning, pain management, digital health biomarker, pain typology, neuropathic, nociceptive

## Abstract

**Background:**

Although it has been well-documented that pain intensity alone is not sufficient to assess chronic pain, the objective pain surface encapsulated in a digital tool might present a major interest in the objective assessment of pain.

**Objective:**

This study aims to determine the potential added value of pain surface measurement by determining the correlation between pain surface and pain intensity in chronic pain patients.

**Methods:**

Two databases from observational prospective and retrospective longitudinal studies including patients with chronic pain were used in this research. Pain intensity was assessed by the Numeric Pain Rating Scale. Pain surface (cm²) and pain typology (neuropathic vs mechanical components) were measured by a specific pain mapping digital tool (PRISMap, Poitiers University Hospital). Patients were asked to draw their pain surface on a computerized tactile interface in a predetermined body (adapted from the patient’s BMI). A color code was used to represent pain intensity (very intense, intense, moderate, and low). Simple linear regression was used to assess the proportion of variance in pain surface explained by pain intensity.

**Results:**

The final analysis included 637 patients with chronic pain. The percentage of variance of the pain surface explained by pain intensity was 1.24% (*R*²=0.0124; 95% CI 0.11%-6.3%). In addition, 424 (66.6%) patients used more than 1 intensity or color, among whom 218 (34.2%) used 2 intensities or colors, 155 (24.3%) used 3 intensities or colors, and 51 (8%) used 4 intensities or colors.

**Conclusions:**

This study showed that pain intensity and pain surface provide complementary and distinct information that would help to improve pain assessment. Two-thirds of the cohort used 2 or more intensities to describe their pain. Combining pain intensity and pain surface should be strongly considered as a means of improving daily practice assessment of patients with chronic pain in primary and secondary care.

**Trial Registration:**

ClinicalTrials.gov NCT02964130; https://clinicaltrials.gov/study/NCT02964130?term=PREDIBACK&rank=2

## Introduction

Pain treatment is considered a fundamental human right [1,2]. Chronic pain, affecting millions of people worldwide [3], represents a devastating health condition impacting psychological and social dimensions and finally leading to decreased quality of life [4-6]. This condition also incurs substantial financial burdens, estimated at US $560-$635 billion in 2010 in the United States alone [7]. The multidimensional nature of chronic pain makes it difficult to treat and challenging to assess. Despite advancements in technology, notably in health care, the gold standard of pain assessment still relies on subjective scales, such as the Visual Analog Scale or the Numerical Rating Scale (NRS), which only captures an individual’s self-reported pain intensity.

Given the subjective and multidimensional nature of pain, alternative assessment methods are needed. A holistic approach to pain evaluation, encompassing multiple dimensions, has been recognized as crucial in both clinical and research settings [8-11]. In a prospective longitudinal study involving 200 patients with chronic pain, we applied machine learning techniques based on 432 variables to develop a comprehensive score called the Multidimensional Clinical Response Index (MCRI) [12]. Our study demonstrated that the MCRI better represents patients’ overall health compared to individual outcome measures. MCRI incorporates pain intensity, quality of life, anxiety and depression, functional disability, and pain surface measurements. With a minimal clinical importance difference of 468 cm², pain surface represents 18.9% of the explained variance of the MCRI, while pain intensity represents 27.6%. Specifically, pain surface measurement, encapsulated in a numerical digital tool, entails asking patients to draw their pain related to pain intensity on the tactile interface so as to capture the surface in cm² [13,14]. While pain surface measurement is promising as a means of objectively assessing pain, further investigation is needed to ascertain its potential added value as a digital health biomarker [9]. To determine the potential added value of pain surface assessment, overlapping information between pain intensity and pain surface has to be determined. A significant correlation and overlap between pain intensity and pain surface would suggest that measuring pain intensity alone is sufficient, while a lack of correlation and overlap would indicate that including pain surface provides additional value in pain assessment.

In addition to pain intensity and pain surface, pain typology has been recognized as a significant end point for pain assessment [15]. Pain typology can be distinguished as neuropathic, mechanical or nociceptive, or nociplastic, where neuropathic is characterized by pain caused by a lesion or disease of the somatosensory nervous system, mechanical or nociceptive pain occurs with a normally functioning somatosensory nervous system, and nociplastic is defined as pain arising from the altered function of pain-related sensory pathways in the periphery and central nervous system [16,17]. To date, the pain typology is discriminated using questionnaires (eg, Douleur Neuropathique 4 [18,19], Leeds Assessment of Neuropathic Symptoms and Signs [20,21], PainDETECT [22]), while Initiative on Methods, Measurement, and Pain Assessment in Clinical Trials recommendations guide the use of PainDETECT [22]. To date, no digital solution offers the capability of objectively determining the proportions of mechanical versus neuropathic pain in patients’ experienced pain. Such information would help the clinician to recommend therapy appropriate to a given patient.

Our study aims to determine the potential added value of pain surface mapping using a specific digital tool in patients presenting with chronic pain. We sought to determine the correlation between pain intensity and pain surface in these patients, to determine the extent to which pain surface contributes to more accurate pain diagnosis, and, as a relevant digital health biomarker, to evaluate treatment efficacy in a more objective way. In addition, the proportions of mechanical and neuropathic pain related to pain surface intensity were assessed.

## Methods

### Study Design

The research paper analyzed data from 2 datasets: the PREDIBACK and the PRISMAP studies.

The PREDIBACK study is a prospective observational, multicentric, longitudinal investigation including patients with persistent spinal pain syndrome after spinal surgery (PSPS-T2), in which clinical outcomes were monitored every 3 months over a period of 1 year. PSPS-T2 is characterized as postoperative chronic pain, following one or several spinal surgeries and persisting beyond the healing process [23].

The PRISMAP study is a retrospective monocentric, observational, longitudinal, and real-life cohort study including all patients with chronic pain having had a multidisciplinary consultation at Poitiers University Hospital (France).

### Ethical Considerations

The PREDIBACK study was registered on ClinicalTrials.gov on November 15, 2016, with the identifier NCT02964130. The study protocol received approval from the National Agency for the Safety of Medicines and Health Products under the identifier 2016-A01144-47 and from a French ethical committee (CPP West III). Prior to enrollment, all study participants were provided with comprehensive information about the study procedures. Their written informed consent was obtained to ensure adherence to ethical standards throughout the PREDIBACK study. Written informed consent was obtained from all patients included in the PRISMAP study before data collection. The protocol was approved by the ethics committee of the Poitiers University Hospital (F20210507150101). This study did not receive any financial support from the industry, and the industry did not participate in data collection or data analysis. In order to achieve the objective of this study, namely, to evaluate the correlations between pain surface parameters and pain intensity at a specific point in time, only data from the initial visit of each patient were used.

### Study Participants

Patients had to be at least 18 years of age and experiencing persistent chronic non-cancer pain to be eligible such as complex regional pain syndrome, persistent spinal pain syndrome types 1 and 2, neuropathic pain, and phantom pain. Patients who were currently or had previously received treatments involving spinal cord, subcutaneous, or peripheral nerve stimulation, or an intrathecal drug delivery system were not included. Individuals with a life expectancy of less than 12 months from the time of enrollment, those who were unable to undergo study assessments or complete questionnaires independently, and individuals belonging to vulnerable populations were not included either. Furthermore, any suspicion of substance abuse that could potentially bias the study results resulted in the noninclusion of the participant.

### Objectives and Outcomes

The aim of this exploratory analysis was to evaluate the discriminant validity of pain surface compared to pain intensity (ie, pain surface index evaluates a different construct compared to pain intensity measures). We evaluated the global pain intensity experienced in the previous 24 hours using a digital NPRS ranging from 0=no pain to 10=the worst pain imaginable [24].

**Figure 1:**
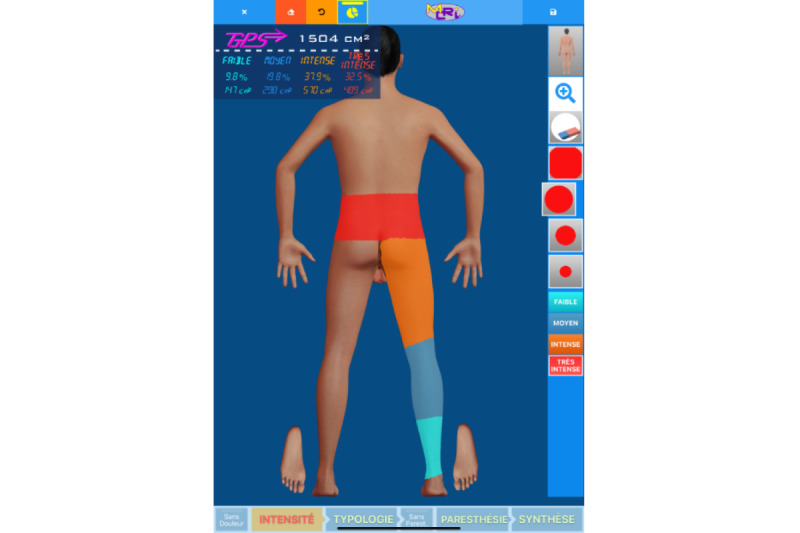
Digital interface used to assess the pain surface of the patient regarding intensity. The image represents a patient with back and leg pain with 4 different intensities (very intense in red, intense in orange, moderate in dark blue, and low in light blue).

**Figure 2:**
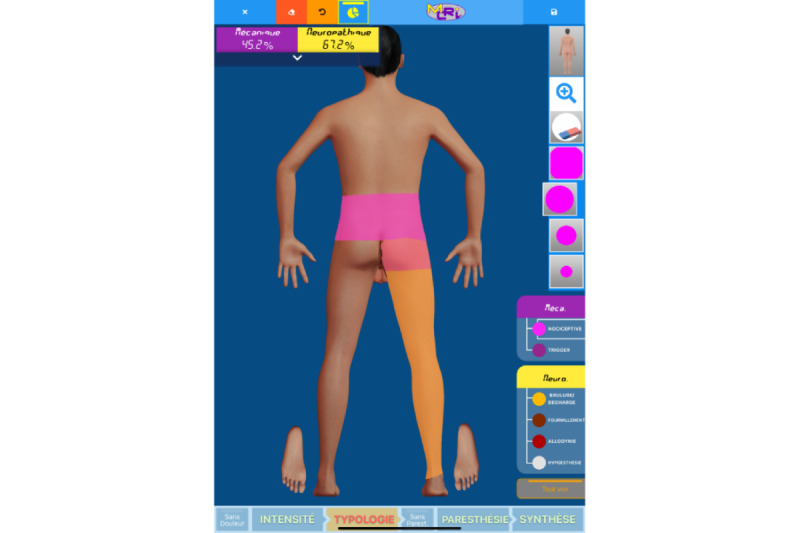
Digital interface used to assess pain typology. The image represents the mechanical (violet) and neuropathic (orange) surfaces. Please note that the intercept between mechanical and neuropathic pain (light pink) represents mixed pain.

The extent of the pain area (measured in cm²) was measured using the PRISMap mapping tool developed in Poitiers University Hospital. Patients drew their pain surface via a touch-sensitive computer interface designed to generate a body adjusted to the patient’s BMI and morphology (Figure 1). The drawings were converted to cm² using a patented process [14]. Pain severity for different pain areas was represented by a color system: light blue for low pain, dark blue for moderate pain, orange for severe pain, and red for very severe pain (Figure 1). The pain surface index consists of the surface in cm² for each intensity multiplied by the weight of the intensity. The weights are the following: 1 for low pain, 2 for moderate pain, 3 for severe pain, and 4 for very severe pain. In addition, pain typology was represented by 2 main typologies (ie, mechanical and neuropathic; Figure 2), violet for mechanical pain with light violet for nociceptive pain and dark violet for trigger pain, and yellow for neuropathic pain with dark yellow for burn or charge pain, brown for tingling, dark red for allodynia, and light grey for hypoesthesia. A nurse was responsible for assisting the patient in differentiating between the various types of pain. In instances where mechanical and neuropathic pain overlapped, the resulting pain was classified as mixed pain (Figure 2).

### Statistical Analysis

The study population was described by the sociodemographic data, diagnosis, and localization of the pain area. Quantitative variables were described using mean and SD or median and IQR. Qualitative variables were described using numbers and percentages. Since this is a correlational study, missing data were not imputed and patients with missing data were removed from the analysis.

In order to evaluate the discriminant validity of the pain surface, pain surface index related to pain intensity, neuropathic pain surface, and mechanical pain surface compared to pain intensity, we used Pearson correlation and reported its 95% CI. We tested whether the correlation between the pain surface index and NPRS was smaller than 1 using a chi-square test comparing the model where the correlation is constrained to 1 and the model with unconstrained correlation.

We also estimated the average variance extracted (AVE) [25] to measure the amount of variance in a construct relative to the amount of variance in “measurement error” by calculating the covariance of pain intensity and pain surface index, and by dividing it by half of the sum of the variance of the 2 measured variables. The results provide the percentage of the common variance between NPRS and pain surface index. An AVE smaller than 0.5 indicates that the majority of the variance of the 2 indicators is not common.

The nonlinear relationship between NPRS and pain surface parameters was graphically investigated using the locally estimated scatterplot smoothing nonparametric model. In this model, the pain surface parameters were transformed using a log transformation (log(surface + 1)) in order to facilitate visual interpretations. In addition, a degree 3 polynomial regression analysis was performed to estimate the cubic relationship between the log-transformed pain surface parameters and NPRS. The *R*^2^ of the models was reported.

In order to justify the need for zone-specific pain intensity evaluation, we evaluated the rate of patients who used more than 1 color to draw their pain surface.

Finally, we compared the pain intensity NPRS score between patients with different predominant pain surface intensities (ie, the intensity representing the color with the highest surface). With this in mind, median NPRS was compared between predominant intensity groups using the Jonckheere trend test, which tests whether NPRS increases as the predominant pain surface intensity increases or not.

We used R software (version 4.2.2; R Foundation for Statistical Computing) to perform the analyses. We used the factor analysis framework from the *lavaan* and *semTools* packages.

## Results

**Figure 3:**
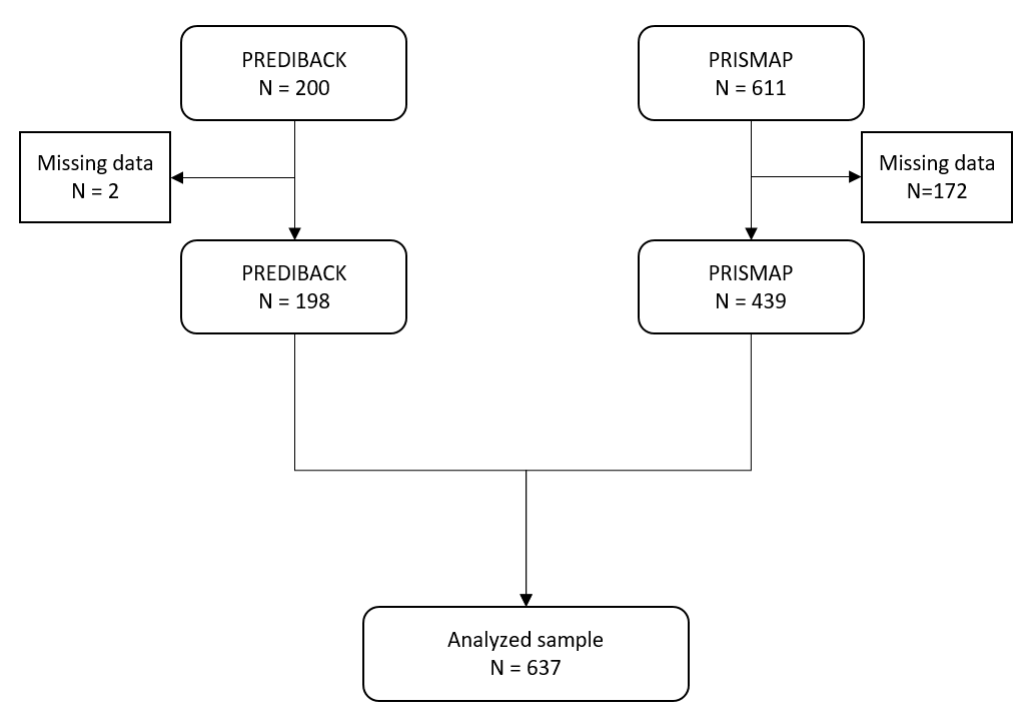
Flowchart of study patients.

We included 200 patients in the PREDIBACK study from 5 French pain centers (Angouleme, Bressuire, La Rochelle, Niort, and Poitiers) between January 2017 and March 2018. In addition, 611 patients were included in the PRISMAP dataset including patients from 3 French pain centers (La Rochelle, Niort, and Poitiers) between January 2018 and January 2023. All in all, 811 patients were included. After removing missing data, 637 patients were analyzed (Figure 3).

Patient characteristics can be found in Table 1. Patients’ mean age was 52.0 (SD 12.6) years, and 356 (55.9%) were female. The pain was located in the back or leg(s) for 366 (57.5%) of the patients and 366 (57.5%) had PSPS-T2.

Data are presented in Table 2 for PREDIBACK, PRISMAP, and full datasets. For the full dataset, the mean NPRS was 6.4 (SD 2.0) points and the mean pain surface was 668.2 (SD 837.6) cm². The very intense surface represented on average 37.1% (SD 36.8%) of the total pain surface, intense surface represented 32.6% (SD 34.3%), moderate surface represented 23.7% (SD 29.8%), and low surface represented 6.7% (SD 17.8%). On average, neuropathic pain surface represented 56.2% (SD 42.2%) and 411.8 (SD 558.8) cm² of the total pain surface, and mechanical pain surface represented 28.0% (SD 30.1%) and 131.2 (SD 175.0) cm². On average, neuropathic pain comprised 33.7% (SD 30.1%) of very intense pain, 28.2% (SD 31.2%) of intense pain, 18.5% (SD 24.7%) of moderate pain, and 5.9% (SD 14.6%) of low pain. On the other hand, mechanical pain surface comprised 38.7% (SD 45.6%) of very intense pain, 33% (SD 42.8%) of intense pain, 19.4% (SD 33.7%) of moderate pain, and 4.2% (SD 17.3%) of low pain.

**Figure 4:**
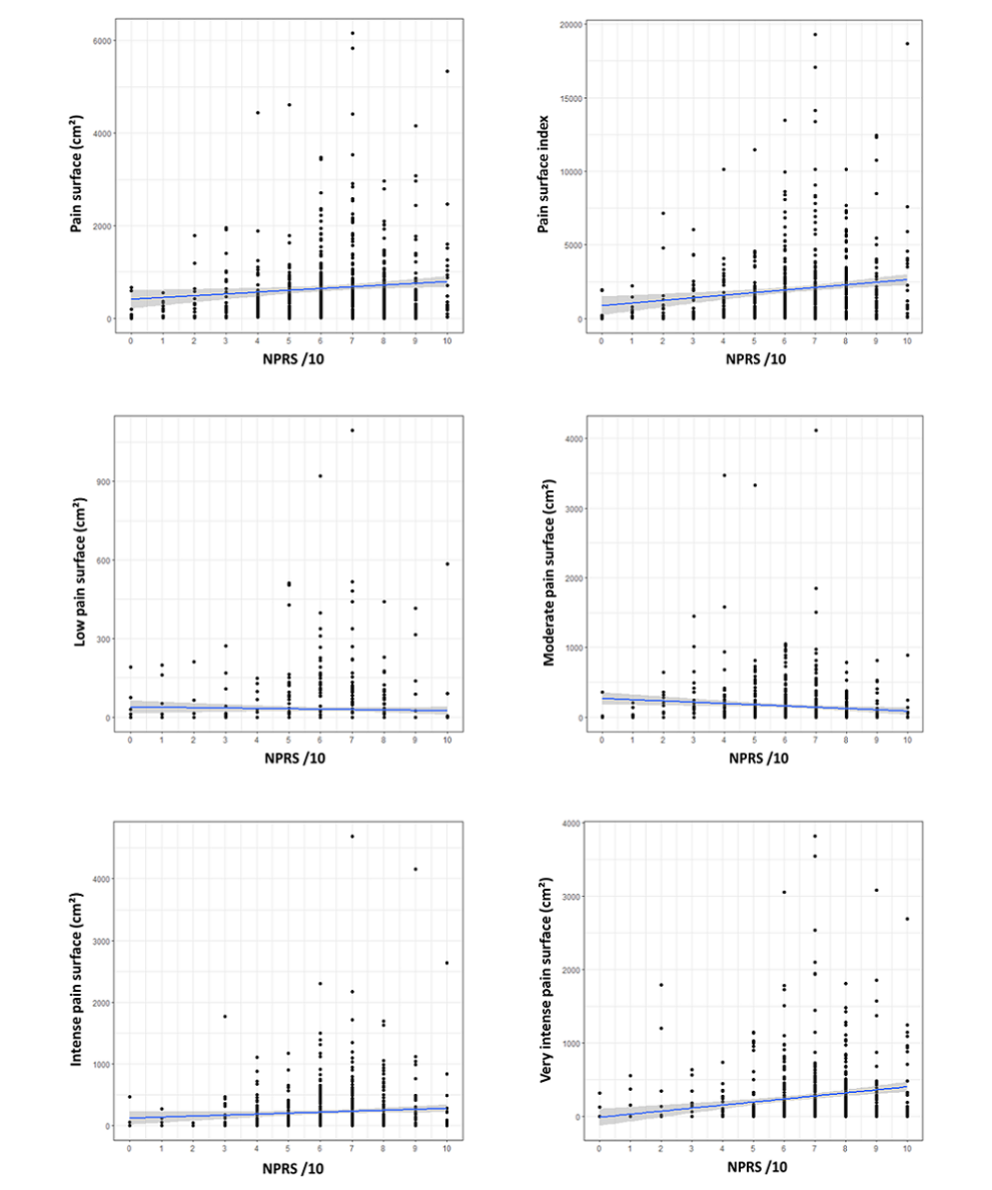
Scatter plot and regression lines (with 95% CI) of the relationship between the different pain surface variables and pain intensity. NPRS: Numeric Pain Rating Scale.

The statistical analysis showed a significant but low Pearson correlation between NPRS and pain surface index (*r*=0.14; 95% CI 0.06-0.21; *P*<.001), with a variance explanation (*R*^2^) of 2%. The coefficient of correlation was significantly different from 1 (*P*<.001) (Table 3). The AVE was 0.0002, indicating a very low percentage of common variance between NPRS and pain surface. Similarly, the correlation between pain surface (cm²) and NPRS was significant but low (*r*=0.09, 95% CI 0.02-0.17; *P*=.04). Figure 4 shows the scatterplot between NPRS and different pain surface parameters.

**Table 1:** Study patient characteristics.

Variables			PREDIBACK dataset (n=198)		PRISMAP dataset (n=439)		Full dataset (N=637)
Age (years), mean (SD)			52.8 (12.5)		51.7 (12.6)		52.0 (12.6)
Sex (male), n (%)			87 (43.9)		194 (44.2)		281 (44.1)
**Pain location, n (%)**							
	Back and leg	190 (96)		127 (28.9)		317 (49.8)	
	Back	7 (3.5)		14 (3.2)		21 (3.3)	
	Leg	1 (0.5)		27 (6.2)		28 (4.4)	
	Face	0 (0)		13 (3)		13 (2)	
	Upper limbs	0 (0)		21 (4.8)		21 (3.3)	
	Groin	0 (0)		15 (3.4)		15 (2.4)	
	Other	0 (0)		222 (50.6)		222 (34.9)	
**Diagnoses, n (%)**							
	PSPS-T2^a^	198 (100)		168 (38.3)		366 (57.5)	
	CRPS^b^	0 (0)		22 (5)		22 (3.5)	
	Neuropathic pain	0 (0)		46 (10.5)		46 (7.2)	
	Radiculopathy	0 (0)		37 (8.4)		37 (5.8)	
	Perineal pain	0 (0)		22 (5)		22 (3.4)	
	Brachial plexus avulsion	0 (0)		8 (1.8)		8 (1.3)	
	Myelopathy	0 (0)		7 (1.6)		7 (1.1)	
	Trigeminal neuralgia	0 (0)		5 (1.1)		5 (0.8)	
	Diabetic neuropathy	0 (0)		5 (1.1)		5 (0.8)	
	Phantom pain	0 (0)		4 (0.9)		4 (0.6)	
	Postherpetic neuralgia	0 (0)		4 (0.9)		4 (0.6)	
	Dorsaligia	0 (0)		3 (0.7)		3 (0.5)	
	Postradiation neuropathy	0 (0)		3 (0.7)		3 (0.5)	
	Thoracic outlet syndrome	0 (0)		3 (0.7)		3 (0.5)	
	Cluster headache	0 (0)		2 (0.5)		2 (0.3)	
	Other	0 (0)		8 (1.8)		8 (1.3)	
	Unknown	0 (0)		92 (21)		92 (14.4)	

^a^PSPS-T2: persistent spinal pain syndrome type 2.

^b^CRPS: complex regional pain syndrome.

**Table 2:** Pain intensity (NPRS^a^), pain surface, and pain typology.

Variables	PREDIBACK dataset (n=198), mean (SD)	PRISMAP dataset (n=439), mean (SD)	Full dataset (N=637), mean (SD)
NPRS	6.1 (1.5)	6.6 (2.2)	6.4 (2.0)
Pain surface (cm²)	686.3 (570.1)	660.1 (933.3)	668.2 (837.6)
Very intense pain surface, cm²; %	213.8 (469.0); 25.0 (34.6)	272.0 (449.3); 42.5 (37.8)	253.9 (455.5); 37.1 (36.8)
Intense pain surface, cm²; %	273.3 (342.7); 40.5 (37.0)	197.3 (428.4); 29.2 (33.0)	220.9 (403.8); 32.6 (34.3)
Moderate pain surface, cm²; %	171.6 (232.4); 29.6 (33.3)	146.5 (376.5); 21.0 (28.1)	154.3 (338.4); 23.7 (29.8)
Low pain surface, cm²; %	27.6 (74.0); 5.0 (13.1)	44.3 (248.4); 7.4 (19.6)	39.1 (210.4); 6.7 (17.8)
Neuropathic pain surface, cm²; %	458.2 (458.3); 61.2 (34.4)	390.9 (598.6); 54.0 (45.3)	411.8 (558.8); 56.2 (42.2)
Very intense neuropathic surface, cm²; %	135.9 (325.5); 24.4 (35.7)	189.0 (417.2); 37.9 (27.2)	172.5 (391.1); 33.7 (30.1)
Intense neuropathic surface, cm²; %	198.3 (309.0); 41.5 (40.3)	100.6 (224.0); 22.2 (26.1)	131.0 (253.4), 28.2 (31.2)
Moderate neuropathic surface, cm²; %	107.4 (183.3); 29.6 (38.0)	70.3 (163.7); 13.5 (15.4)	81.8 (170.0); 18.5 (24.7)
Low neuropathic surface cm²; %, mean (SD)	16.6 (51.2); 4.6 (13.3)	30.1 (95.2); 6.6 (15.2)	25.9 (84.1); 5.9 (14.6)
Mechanical pain surface, cm²; %	143.4 (167.8); 28.6 (30.2)	125.7 (178.2); 27.7 (30.1)	131.2 (175.0); 28.0 (30.1)
Very intense mechanical surface, cm²; %	44.3 (100.1); 26.6 (40.8)	57.0 (97.0); 44.2 (47.%)	53.1 (98.0); 38.7 (45.6)
Intense mechanical surface, cm²; %	56.8 (108.8); 37.0 (44.3)	34.6 (83.6); 31.2 (42.1)	41.5 (92.2); 33.0 (42.8)
Moderate mechanical surface, cm²; %	34.0 (63.2); 31.7 (43.3)	23.6 (71.0); 13.9 (28.4)	26.8 (68.7); 19.4 (33.7)
Low mechanical surface, cm²; %	8.4 (49.7); 4.1 (16.9)	10.4 (60.6); 4.3 (17.5)	9.8 (57.4); 4.2 (17.3)

^a^NPRS: Numeric Pain Rating Scale.

**Table 3:** Correlation of pain surface and pain typology with NPRS^a^.

Correlation with NPRS	ρ (95% CI)	AVE^b^
Surface	0.09^c^ (0.015-0.169)	0.00045
Pain surface index	0.14^d^ (0.062-0.214)	0.00022
Low pain surface	–0.006 (–0.08 to 0.07)	0.00012
Moderate pain surface	–0.10^c^ (–0.18 to –0.02)	0.0012
Intense pain surface	0.07 (–0.005to 0.15)	0.00073
Very intense pain surface	0.18^d^ (0.10-0.26)	0.0016
Mechanical pain surface	0.03 (– 0.095 to 0.153)	0.0091
Neuropathic pain surface	–0.07 (–0.19 to 0.05)	0.0076

^a^NPRS: Numeric Pain Rating Scale.

^b^AVE: average variance extracted.

^c^*P*<.05.

^d^*P*<.001.

**Figure 5:**
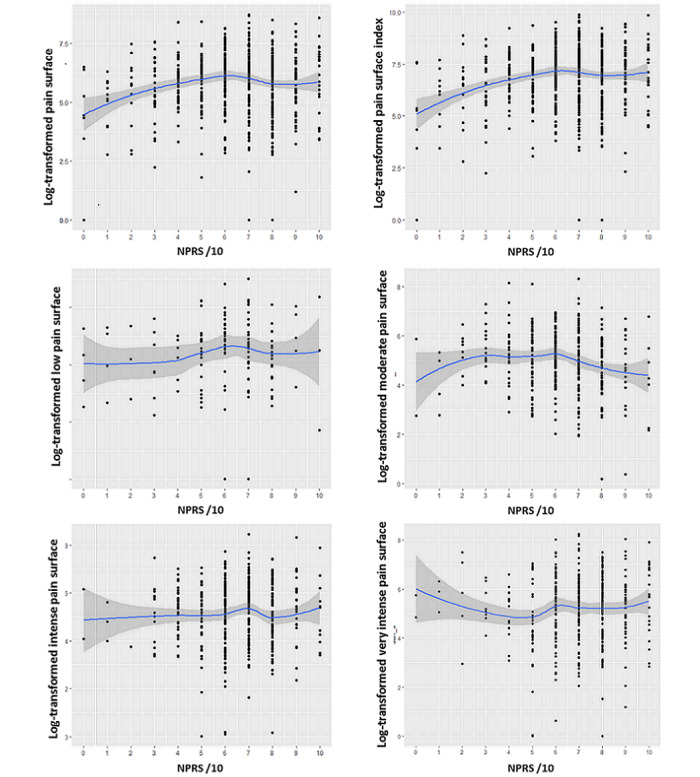
Smoothed scatter plot (with SE) of the relationship between the different log-transformed pain surface variables and pain intensity. NPRS: Numeric Pain Rating Scale.

Regarding the nonlinear relationship between the NPRS and log-transformed data, the locally estimated scatterplot smoothing regression fits for different pain surface parameters are presented in Figure 5. Pain surface (area and index) increased with NPRS increase when NPRS was inferior to 6 (ie, low and moderate pain), whereas no other trend was observed. The cubic regression analysis estimating the relationship between the log-transformed pain surface index (dependent variable) and a 3-degree polynomial of NPRS score showed statistical significance for the intercept (6.9; 95% CI 6.8-7.0; *P*<.001), for the first-degree coefficient (7.1; 95% CI 4.4-9.7; *P*<.001) and the second-degree coefficient (–3.3; 95% CI –6.0 to –0.6; *P*=.015), but not for the third-degree coefficient (–1.5; 95% CI –4.1 to 1.1; *P*=.28). The estimated R-squared of the cubic model was 5.1%. Similarly, the cubic regression analysis describing the log-transformed pain surface based on pain surface showed statistical significance for the intercept (5.9; 95% CI 5.8-6.0; *P*<.001), for the first-degree coefficient (4.2; 95% CI 1.7-6.7; *P*=.001) and the second-degree coefficient (–3.8; 95% CI –6.3 to –1.3; *P*=.003), but not for the third-degree coefficient (–1.5; 95% CI –4.6 to 0.4; *P*=.10). The estimated R-squared of the cubic model was 3.4%.

During the assessment of pain surface related to pain intensity, 3 (0.5%) patients had no pain during the assessment, while 210 (33%) used 1 color, 218 (34.2%) used 2 colors, 155 (24.3%) used 3 colors, and 51 (8%) used the 4 available colors. In summary, 33% used 1 color, whereas 66.6% used 2 or more colors.

The mean NPRS was 7.2 (SD 1.8) for patients with a predominant very intense pain surface (n=242; 38%), 6.4 (SD 1.8) for patients with a predominantly intense surface (n=207; 32.5%), 5.4 (SD 1.9) for patients with a predominantly moderate pain surface (n=154; 24.2%), and 5.1 (SD 2.7) for patients with a predominantly low pain surface (n=31; 4.9%). This observed decrease in NPRS based on the predominant pain surface was significant (*P*<.001).

## Discussion

### Principal Findings

By investigating correlations between NPRS and different pain surface parameters, our results showed very low variance explanation (1.9%) and AVE (0.002), indicating that NRPS and pain surface did not provide overlapping information. In addition, two-thirds of the 637 patients used more than 1 color or intensity to characterize their pain, indicating that a single global measure of pain intensity was not sufficient to define pain perception.

Pain intensity score was initially developed to quantify acute pain, notably in primary care. While the strong relevance of pain intensity measures has been demonstrated in acute pain assessment, pain intensity alone provides limited information in chronic pain populations. Among the most widely used pain intensity scores, it was recently reported that the NRS and Visual Analog Scale were not consistent in their evaluations of pain intensity [26]. Initiative on Methods, Measurement, and Pain Assessment in Clinical Trials consensus and literature reviews have converged to recommend the NRS (0-10) to assess pain intensity in chronic pain [15,27,28]. In addition to pain intensity, the localization of painful body areas has been identified as a major outcome in pain assessment [15]. To date, body area is assessed by standardized body chart as in the Brief Pain Inventory [29], painDETECT [22,30], or the Leeds Assessment of Neuropathic Symptoms and Signs [20,21] questionnaires. In order to improve descriptive body charts, the PRISMap digital tool was developed to offer the possibility for the first time to deliver objective measurements by measuring the pain surface in a given individual [14]. Furthermore, the avatar displayed on the pain-mapping digital tool is generated based on patients’ morphology, including height and body mass, so as to adjust the total body surface, thereby ensuring personalized and precise pain surface assessment. Based on 637 patients, our study showed that global pain intensity and pain surface provide distinct information, where global pain intensity translates pain perception and pain surface creates a graphical representation of pain localization at 4 different pain intensity levels (very intense, intense, moderate, and low) via numerical metrics. Furthermore, two-thirds of the patients used at least 2 colors to describe their pain via the mapping digital tool, whereas only 1 global pain intensity score would appear to induce misinterpretation and inaccuracy of pain perception evaluation. In a recent prospective observational study including 200 patients with PSPS-T2, we demonstrated that pain surface can be considered as a predictor of therapy outcomes, while large pain surface is a negative predictor and small surface a positive predictor [31]. By investigating dementia risk in 354,943 individuals, Zhao et al [32] recently reported that individuals presenting with multisite chronic pain were associated with higher dementia risk, faster cognitive impairments, and hippocampal atrophy. The single-site or multisite nature of chronic pain should also be considered to improve pain evaluation, especially in vulnerable populations. In clinical practice, both pain intensity and pain surface mapping should be provided to improve pain assessment, especially so as to determine objective pain treatment efficacy and to properly reinterpret patient outcomes.

Our digital tool is able to objectively determine which part of the global pain surface is mechanical pain and which is neuropathic pain by providing specific neuropathic and mechanical pain surfaces related or not to pain intensity. In addition, neuropathic pain can be discriminated into 4 categories: burning or charge, tingling, allodynia, and hypoesthesia sensation (Figure 2). The capacity to determine the proportion of pain typology with a dedicated digital tool would help the primary care service to steer the patient in a specific pathway with specific therapies that could help to prevent the chronification process. Pain typology can also provide an important added value in the assessment of therapeutical tools, correlating efficacy. By collecting nociplastic pain in addition to neuropathic and mechanical pain, the digital tool would offer more information to accurately assess pain in the future [16,17].

From a practical perspective, pain mapping assessment will provide objective measurement of pain surface related to pain intensity and pain typology with an examination time of less than 5 minutes per patient. The digital mapping tool, today primarily dedicated to clinical research, could have a significant benefit for all practitioners in primary and secondary care units. Ultimately, educational procedures would help to train patients to use the pain mapping digital tool at home in telemedicine or prior to a medical or paramedical pain clinic. In addition, the capacity of the digital tool to deliver objective measurements will help to evaluate different therapies’ efficacy for the patient, for the practitioner, and for the regulatory health authorities tasked with calculating reimbursement. Thereby, therapies including topic, transcutaneous electrical nerve stimulation, spinal cord stimulation [33,34], peripheral nerve stimulation [35], manual therapy, and complementary approaches would benefit from this digital tool.

### Conclusions

Our study showed that pain intensity and pain surface were not correlated, indicating the existence of complementary information between the 2 assessments. In addition, the digital tool demonstrated that two-thirds of the study patients with chronic pain used 2 or more intensities or colors to characterize their pain, thereby underscoring the need to provide more than 1 pain intensity measurement. Our digital tool is also able to provide a quantitative assessment of the neuropathic and mechanical or nociceptive nature of pain. All in all, our results indicate that pain intensity, pain surface, and pain typology improve pain assessment and can be considered as a new objective digital biomarker for pain assessment. This new generation of digital tools will be beneficial in primary and secondary care, and at home for the patient in the near future.

## Abbreviations

AVE: average variance extracted
MCRI: Multidimensional Clinical Response Index
NPRS: Numeric Pain Rating Scale
NRS: Numerical Rating Scale
PSPS-T2: persistent spinal pain syndrome type 2

## Data Availability

The data presented in this study are available on request from the corresponding author due to a restrictive access institution policy.
